# Attitudes and beliefs of the French public about schizophrenia and major depression: results from a vignette-based population survey

**DOI:** 10.1186/1471-244X-13-313

**Published:** 2013-11-20

**Authors:** Matthias C Angermeyer, Aurélie Millier, Cécile Rémuzat, Tarek Refaï, Mondher Toumi

**Affiliations:** 1 Center for Public Mental Health, Gösing am Wagram, Austria; 2 Department of Public Health and Clinical and Molecular Medicine, University of Cagliari, Cagliari, Italy; 3 Creativ-Ceutical, Paris, France; 4 Creativ-Ceutical, Tunis, Tunisia; 5 University of Lyon I, Lyon, France

## Abstract

**Background:**

In their study ‘Mental Health in the General Population: Images and Realities’ Jean-Luc Roelandt et al. found a huge divide between the French public’s conceptualizations of insanity and depression. The study aims to examine whether such differences can be replicated using modern operationalized diagnostic criteria for schizophrenia and major depressive disorder.

**Methods:**

In 2012, an online survey was conducted using a representative sample drawn from the adult French population (N = 1600). After presentation of a case-vignette depicting a person with either schizophrenia or major depressive disorder a fully structured interview was carried out.

**Results:**

Despite some similarities marked differences between both disorders emerge regarding beliefs and attitudes. While respondents presented with the schizophrenia vignette more frequently defined symptoms as the expression of an illness with a stronger biological component and a less favorable prognosis, demanding psychiatric treatment, respondents presented with the depression vignette considered the occurrence of symptoms more frequently as the consequence of current psychosocial stress, benefitting not only from established but also from alternative treatments. People with schizophrenia were more frequently perceived as unpredictable and dangerous, there was a stronger need to separate one-self from them, they were more frequently met with fear and less frequently reacted to with pro-social feelings, and they also faced more rejection.

**Conclusions:**

The French public draws a clear line between schizophrenia and major depressive disorder. This applies equally to beliefs about both disorders and to attitudes towards the persons afflicted. There is a need for interventions trying to reduce existing misconceptions in order to improve the care of patients.

## Background

In their ground-breaking study ‘Mental Health in the General Population: Images and Realities’ Jean-Luc Roelandt et al. [1,2] provided a comprehensive description of how mental illness is perceived by the general public in France. Using the concept of social representations as a theoretical framework, they explored the public’s notion of the ‘insane’ , the ‘mentally ill’ , and the ‘depressive’. They found that the public draws a clear line between the representation of the ‘insane’ on one hand and the ‘depressive’ on the other hand. While insane people were described as abnormal, irresponsible, socially excluded, far from being curable, and to be treated against their will by psychotropic drugs and psychiatric hospitalization, depressive people were perceived as more familiar, suffering, and curable. Moreover, over 75% of respondents associated the words ‘insane’ with violent and dangerous behaviors while the term ‘depressive’ was associated with sadness, isolation, and suicide.

Stimulated by Roelandt et al.’s work we set out to also investigate the French public’s perception of mental illness, this time studying it from a different theoretical and methodological angle. Instead of employing words used in everyday language to denote mental illness (‘insane’ , ‘depressive’) as stimulus, we presented the interviewees with vignettes depicting a person suffering from symptoms of either schizophrenia (which was assumed to correspond most closely to what is understood by the term ‘insane’) or major depressive disorder. Thus, while in Roelandt et al.’s study the public’s associations to mundane labels were assessed, in our study reactions to descriptions of pathological behavior fulfilling diagnostic criteria used in psychiatry were examined.

Vignette-based studies conducted in other countries indicate that despite some similarities there are marked differences between the two disorders as concerns public beliefs and attitudes. Schizophrenia was found to be seen as an illness with a strong biological component and a rather unfavorable prognosis, demanding psychiatric treatment. Major depressive disorder, in contrast, was considered more as a consequence of the exposure to psycho-social stress, benefitting mostly from psychotherapy and alternative treatments. Moreover, people with schizophrenia were more frequently perceived as unpredictable and dangerous. They were more frequently met with fear and less frequently reacted to with pro-social feelings. People with schizophrenia also did face more rejection than those with depression [3-5].

In this paper, we will present findings on the public’s knowledge and beliefs about schizophrenia and major depressive disorder as well as on public attitudes towards people suffering from these disorders. For the study of the public’s knowledge and beliefs, Anthony Jorm’s [6] concept of mental health literacy will serve as the theoretical framework. Mental health literacy refers to ‘knowledge and beliefs about mental disorders which aid their recognition, management or prevention’ and includes ‘the ability to recognize specific disorders; knowing how to seek mental health information; knowledge of risk factors and causes, of self-treatments, and of professional help available’ [7]. This knowledge may play an important role in seeking help for mental health problems [8].

For the study of attitudes we used as the theoretical framework the stigma concept developed by Bruce Link and Jo Phelan [9,10]. According to the authors, stigma exists when the following interrelated components converge: (1) people distinguish and label human differences; (2) dominant cultural beliefs link labeled persons to undesirable characteristics that form the stereotype; (3) labeled persons are seen as an out-group, as ‘them’ and not ‘us’; (4) stereotyping and separating evoke negative emotions; and (5) labeled persons experience loss of status and discrimination. One form of discrimination is the overt discrimination directed towards a person with a mental disorder, such as rejecting their job application or refusing to rent them an apartment. In research, this is most frequently measured by desire for social distance.

The aim of our study is to examine to what extent Roelandt et al.’s findings can be replicated using case-vignettes with the description of mental disorders fulfilling modern operationalized diagnostic criteria. We want explore potential differences in the French public’s knowledge and beliefs about schizophrenia and major depressive disorder. More specifically, we want to determine what differences exist between both disorders with regard to recognition, causal attributions, expected prognosis, help-seeking preferences, and treatment beliefs. Moreover, we want to examine to what extent public attitudes towards people affected by these disorders differ and what differences exist with regard to stereotypes, separation of ‘us’ from ‘them’ , emotional reactions, and desire for social distance.

## Methods

### Survey

Persons aged 16–65 years old, of French nationality, were drawn from an established market research panel. The panel is recruited and managed under the highest standards (‘MRA Verified’ seal, adhering to CASRO and ESOMAR guidelines). Persons were contacted by email and invited to participate in an on-line survey between January and March 2012. If a person did not respond to the initial contact, he or she was contacted again three days later. Recruitment continued until 1600 interviews were obtained. Participants were aware that their responses would be used for scientific research, specifically commissioned by the authors. To ensure that the sample was representative of the general adult population of France, sampling was stratified for place of residence, gender, age, and family status. In total, 1,600 persons were interviewed. The socio-demographic characteristics of the sample reflect fairly well the socio-demographic composition of the general population in France [11]: 50% were men (France: 50.6%); 17.5% of respondents were 16–24 years old, 20.2% 25–34 years old, 22.1% 35–44 years old, 21.3% 45–54 years old, and 18.9% 55–65 years old (France: 17.1%, 19.1%, 20.9%, 21.1%, and 21.7%, respectively). 43.1% of respondents were single (France: 45.8%). The educational level of respondents was as follows: 17.3% CAP/BEP, 21.7% bachelor, and 61.0% superior or other (France: 23.4%, 17.1%, and 59.5%, respectively). Informed consent was considered to have been given when individuals agreed to complete the interview.

### Interview

The fully-structured interview had originally been developed in Germany and had been successfully employed there in several surveys [4,12]. For use in this study, it was translated into French following the guidelines of WHO [13]. At the beginning of the interview respondents were presented with a *vignette* of a diagnostically unlabeled psychiatric case history, depicting a case of either schizophrenia or major depressive disorder. The symptoms described in the vignettes that had originally been prepared for the German surveys fulfilled the criteria of DSM-III-R for the respective disorder [14]. Each vignette had been independently rated by five experts on psychopathology masked to actual diagnosis, providing confirmation of the correct diagnosis for each case history. In order to be able to compare our findings with those from Germany we decided to use the same vignettes. The sex of the individual presented in the vignettes was randomly varied. Respondents were randomly allocated to receive either the vignette depicting schizophrenia or the vignette depicting major depressive disorder.

### Measures of beliefs about schizophrenia and major depressive disorder

Following the presentation of the vignette, respondents were asked whether the problem depicted in the vignette represented in their eyes ‘a mental illness in the medical sense’ or not. Next, respondents’ *causal attributions* were elicited with a list of twelve possible causes, each of which had to be rated on a five-point Likert scale anchored with 1 = ‘certainly a cause’ and 5 = ‘certainly not a cause’. Three items each were referencing either current psychosocial stress (‘stressful life-event’ , ‘work-related stress’ , ‘problems with partner and family’), childhood adversities (‘grown up in a broken home’ , ‘lack of parental affection’ , ‘childhood sexual abuse’), biogenetic causes (‘chemical imbalance in the brain’ , ‘brain disease’ , ‘heredity’), and intra-psychic causes (‘immoral life style’ , ‘weak will’ , ‘unconscious conflict’). Respondents who endorsed the two points on either side of the mid-point of the scale (‘undecided’) were grouped together into the categories ‘a cause’ or ‘not a cause’ , respectively.

With the help of the following items the *prognosis* anticipated by respondents was assessed: ‘The person will never get over it completely’; ‘The person will never be able to make important decisions alone’; ‘The person is going to decline a priori’; ‘The person will never be able to perform regular professional obligations’. The items had to be rated on a five-point Likert scale anchored with 1 = ‘totally agree’ and 5 = ‘totally disagree’. Respondents who endorsed the two points on either side of the mid-point of the scale (‘undecided’) were grouped together into the categories ‘agree’ or ‘disagree’ , respectively.

Considering attitudes towards treatment, a distinction was made between health care providers and treatment methods. *Help-seeking recommendations* were assessed using a catalogue of the following sources of help: psychiatrist, psychotherapist, general practitioner, health practitioner, priest, self-help group, the Internet, and confidant. The respondents were asked to indicate their endorsement or rejection of each source of help, using a five-point Likert scale ranging from ‘would strongly recommend’ to ‘would not recommend at all’ plus a ‘don’t know’ category. Respondents who endorsed the two points on either side of the mid-point of the scale (‘undecided’) were grouped together into the categories ‘recommend’ or ‘advise against’, respectively.

Using a five-point scale ranging from ‘would strongly recommend’ to ‘would not recommend at all’ (plus ‘don’t know’) respondents were asked to provide their *treatment recommendations*, offering a list of six different treatment methods, three representing established forms of psychiatric treatment (psychotropic medication, psychotherapy, relaxations techniques) and three ‘alternative’ treatment modalities (natural remedies, mediation, acupuncture). The response categories were combined in the same way as described for help-seeking recommendations.

### Measure of attitudes towards people with mental disorders

After the assessment of respondents’ mental health literacy, questions on their attitudes towards the person depicted in the vignette were asked. According to Hayward and Bright [15], the four most important *stereotypes* about mental illness are that people with mental disorders are unpredictable, that they are dangerous, that they are responsible for their illness, and that mental illnesses are hard to treat. Agreement or disagreement with these stereotypes was recorded with the help of a five-point Likert scale, anchored with 1 = ‘fully agree’ and ‘5’ ‘totally disagree’.

The *tendency to separate oneself from the person in the vignette* was measured by means of the items ‘This person is different from others’ and ‘Basically we are all sometimes like this person. It’s just a question how pronounced this state is’. Agreement or disagreement with these stereotypes was recorded with the help of a five-point Likert scale, anchored with 1 = ‘fully agree’ and ‘5’ ‘totally disagree’.

*Emotional reactions* to the person described in the vignette were assessed by means of nine five-point Likert-scaled items, anchored with 1 = ‘applies completely’ and 5 = ‘does not apply at all’ , representing the main three dimensions fear, anger, and pro-social reactions: ‘I feel the need to help him/her’ , ‘I feel pity’ , ‘I feel uncomfortable’ , ‘He/she makes me feel insecure’ , ‘He/she scares me’ , ‘I feel sympathy for him/her’ , ‘I feel annoyed by him/her’ , ‘I react angrily’ , ‘I am amused by something like that’. Respondents who endorsed the two points on either side of the mid-point of the scale (‘undecided’) were grouped together to the categories ‘agree’ , or ‘disagree’ , respectively [16].

For the assessment of respondents’ *desire for social distance* we used the scale developed by Link et al [17]. This scale encompasses the following social situations: rent a room, work together, have as a neighbor, let take care of a little child, have marry into family, introduce to friends, recommend for a job. With the help of a five-point Likert scale respondents could indicate to what extent they were willing or unwilling to engage in the proposed relationships. Respondents who endorsed the two points on either side of the mid-point of the scale (‘undecided’) were grouped together into the categories ‘accept’ , or ‘reject’ , respectively.

### Statistical analysis

In order to examine whether respondents reacted differently with regard to the schizophrenia vignette and the depression vignette, we calculated separate multinomial logit regressions for each item. To adjust for potential differences between samples for the influence of demographic factors, the regression analyses controlled for respondents’ gender, age, and educational attainment. To illustrate the magnitude of differences between the subsamples presented with the schizophrenia or the depression vignette, discrete probability changes were calculated for all items and each response category with control variables held at their means for the whole sample. A discrete change coefficient is the difference in the predicted probability of a given outcome between both subsamples; it serves as an indicator of the effect size of the change. Ninety-five percent confidence intervals were computed with the delta method. The calculation of probability changes was carried out using SAS 9.3 [18]. To make adjusted predictions comparable to unadjusted survey results, probabilities and discrete changes are multiplied by 100 and can be read as percentage of respondents choosing any answer category. Tables 1, 2, 3, 4, 5, 6, 7, 8 and 9 show, separately for schizophrenia and major depressive disorder, the raw percentage and the predicted probability for each item/answer category plus the difference in probabilities between both disorders and the confidence interval for this difference.

**Table 1:** Labeling of schizophrenia and major depression

	**Schizophrenia**		**Major depression**			
	**Raw %**	**Adj. %**	**Raw %**	**Adj. %**	**Change**	**95% CI**
Mental illness	78.0	78.8	58.1	59.3	−19.5	[−23.9 ; -15.0]
No mental illness	7.6	7.4	20.6	20.1	12.7	[9.4 ; 16.0]
Don’t know	14.4	13.8	21.2	20.5	6.7	[0.8 ; 12.7]

**Table 2:** Causal beliefs regarding schizophrenia and major depression

		**Schizophrenia**		**Major depression**			
		**Raw %**	**Adj. %**	**Raw %**	**Adj. %**	**Change**	**95% CI**
Brain disease	Agree	45.4	45.0	17.0	16.6	−28.4	[−32.9 ; -23.9]
	Undecided	25.2	24.9	19.9	19.3	−5.6	[−9.7 ; -1.4]
	Disagree	29.4	30.1	63.1	64.0	33.9	[30.0 ; 37.8]
Hereditary factors	Agree	26.2	24.0	11.4	10.2	−13.8	[−17.8 ; -9.9]
	Undecided	23.4	23.8	17.6	17.5	−6.3	[−10.4 ; -2.3]
	Disagree	50.4	52.1	71.0	72.4	20.3	[16.5 ; 23.9]
Disturbance of brain metabolism	Agree	50.9	51.7	24.0	24.2	−27.5	[−32.2 ; -22.8]
	Undecided	25.2	23.6	24.5	22.6	−1.0	[−5.2 ; 3.1]
	Disagree	23.9	24.7	51.5	53.3	28.6	[24.1 ; 33.1]
Troubles in family/ partnership	Agree	43.5	44.2	78.1	78.4	34.2	[29.6 ; 38.9]
	Undecided	30.4	31.1	15.3	15.4	−15.7	[−20.0 ; -11.3]
	Disagree	26.1	24.7	6.6	6.1	−18.6	[−20.6 ; -16.5]
Negative life events	Agree	64.9	66.0	79.6	80.5	14.5	[10.1 ; 18.8]
	Undecided	21.4	20.6	12.4	11.7	−8.9	[−12.5 ; -5.2]
	Disagree	13.7	13.5	8.0	7.8	−5.7	[−16.0 ; -1.7]
Stress at work	Agree	47.0	49.3	79.7	81.6	32.3	[26.3 ; 38.2]
	Undecided	28.5	25.8	12.3	10.6	−15.2	[−19.7 ; -10.7]
	Disagree	24.5	24.9	8.0	7.8	−17.1	[−19.1 ; -14.9]
Broken home	Agree	21.9	20.1	17.3	15.6	−4.5	[−8.1 ; -0.8]
	Undecided	27.0	26.1	21.1	20.0	−6.1	[−10.1 ; -2.0]
	Disagree	51.1	53.8	61.6	64.4	10.6	[6.6 ; 14.5]
Lack of parental affection	Agree	24.4	24.4	24.0	24.2	−0.2	[−4.5 ; 4.0]
	Undecided	32.6	33.1	29.5	29.8	−3.3	[−7.9 ; 1.2]
	Disagree	43.0	42.4	46.5	46.1	3.7	[−0.4 ; 7.7]
Sexual abuse in childhood	Agree	24.4	24.2	24.5	24.3	0.1	[−4.1 ; 4.4]
	Undecided	32.6	32.8	23.2	23.5	−9.3	[−13.7 ; -4.9]
	Disagree	43.0	43.0	52.3	52.1	9.1	[5.1 ; 13.2]
Immoral life style	Agree	15.1	12.3	13.0	10.4	−1.9	[−4.8 ; 1.0]
	Undecided	18.9	19.3	19.5	19.7	0.4	[−3.5 ; 4.4]
	Disagree	66.0	68.4	67.5	69.9	1.5	[−2.3 ; 5.3]
Weak will	Agree	34.5	32.8	24.1	22.6	−10.2	[−14.6 ; -5.9]
	Undecided	23.0	23.6	26.5	26.7	3.1	[−1.2 ; 7.4]
	Disagree	42.5	43.5	49.4	50.7	7.2	[3.0 ; 11.3]
Unconscious conflict	Agree	53.4	54.1	51.5	52.2	−1.9	[−6.8 ; 3.0]
	Undecided	27.0	25.3	27.6	26.0	0.7	[−3.6 ; 4.8]
	Disagree	19.6	20.6	20.9	21.8	1.2	[−4.3 ; 6.7]

**Table 3:** Expected prognosis of schizophrenia and major depression

		**Schizophrenia**		**Major depression**			
		**Raw %**	**Adj. %**	**Raw %**	**Adj. %**	**Change**	**95% CI**
The person will never get over it completely	Agree	26.0	25.3	17.9	17.2	−8.1	[−12.1 ; -4.1]
	Undecided	33.4	34.1	22.0	22.4	−11.7	[−16.2 ; -7.2]
	Disagree	40.6	40.7	60.1	60.5	19.8	[15.9 ; 23.7]
The person will never be able to make important decisions alone	Agree	17.6	17.1	8.9	8.3	−8.8	[−12.2 ; -5.5]
	Undecided	33.5	33.6	15.7	15.3	−18.3	[−22.5 ; -13.9]
	Disagree	48.9	49.3	75.4	76.4	27.1	[23.7 ; 30.5]
The person will never be able to perform regular professional obligations	Agree	18.1	17.3	6.6	6.0	−11.3	[−14.7 ; -7.9]
	Undecided	31.6	29.9	17.5	15.8	−14.1	[−18.3 ; -10.0]
	Disagree	50.3	52.8	75.9	78.2	25.4	[22.1 ; 28.8]
The person will always be dependent on others’ help	Agree	12.4	11.4	6.1	5.5	−5.9	[−8.8 ; -3.0]
	Undecided	35.6	36.2	19.9	19.8	−16.4	[−20.8 ; -11.8]
	Disagree	52.0	52.5	74.0	74.7	22.2	[18.7 ; 25.7]

**Table 4:** Help-seeking recommendations for schizophrenia and major depression

		**Schizophrenia**		**Major depression**			
		**Raw %**	**Adj. %**	**Raw %**	**Adj. %**	**Change**	**95% CI**
Psychiatrist	Agree	81.0	82.0	58.6	60.5	−21.5	[−34.4 ; -8.7]
	Undecided	8.5	7.7	19.7	18.1	10.4	[7.7 ; 13.2]
	Disagree	6.6	6.4	17.3	16.9	10.5	[7.9 ; 13.2]
	Don’t know	3.0	3.9	4.4	4.5	0.6	[0.0 ; 1.2]
Psychotherapist	Agree	76.8	78.1	68.2	70.0	−8.1	[−22.1 ; 5.8]
	Undecided	12.4	11.7	18.0	17.1	5.4	[3.2 ; 7.6]
	Disagree	7.2	6.8	9.8	9.2	2.4	[0.8 ; 4.0]
	Don’t know	3.6	3.4	4.0	3.7	0.3	[−0.2 ; 0.8]
General practitioner	Agree	74.9	76.8	82.7	83.8	7.0	[−15.9 ; 29.9]
	Undecided	13.4	13.4	9.8	9.6	−3.8	[−42.4 ; 34.8]
	Disagree	8.9	9.2	6.3	6.3	−2.9	[−25.0 ; 19.3]
	Don’t know	2.9	0.6	1.2	0.3	−0.3	[−6.6 ; 5.9]
Practitioner of complementary medicine	Agree	17.1	14.4	23.9	20.6	6.2	[−3.0 ; 15.5]
	Undecided	19.2	18.1	23.7	22.8	4.7	[−1.0 ; 10.4]
	Disagree	51.1	55.0	41.3	45.2	−9.8	[−17.3 ; -2.3]
	Don’t know	12.6	12.5	11.1	11.3	−1.2	[−2.5 ; 0.3]
Pastor/priest	Agree	9.5	9.3	13.2	12.9	3.6	[−9.9 ; 17.0]
	Undecided	17.7	17.4	18.5	18.1	0.7	[−18.1 ; 19.6]
	Disagree	55.8	57.1	53.5	54.9	−2.2	[−9.9 ; 5.6]
	Don’t know	17.0	16.2	14.8	14.1	−2.1	[−3.5 ; -0.8]
Health cure	Agree	9.1	9.5	17.6	18.6	9.1	[3.2 ; 15.0]
	Undecided	18.0	16.1	31.7	29.0	12.9	[−0.4 ; 26.1]
	Disagree	53.4	56.3	37.6	40.3	−16.0	[−24.9 ; -7.1]
	Don’t know	19.5	18.1	13.0	12.1	−6.0	[−7.3 ; -4.7]
Confidant	Agree	59.9	58.2	76.7	75.6	17.4	[2.5 ; 32.4]
	Undecided	22.7	23.4	15.0	15.5	−7.9	[−11.2 ; -4.6]
	Disagree	13.5	14.2	6.5	6.9	−7.3	[−9.5 ; -5.1]
	Don’t know	3.9	4.3	1.8	1.9	−2.4	[−2.8 ; -1.8]
Self-help group	Agree	50.2	50.3	46.2	46.3	−4.0	[−11.5 ; 3.5]
	Undecided	24.1	24.4	31.5	31.6	7.2	[2.2 ; 12.2]
	Disagree	13.8	15.7	12.4	14.2	−1.5	[−6.3 ; 3.4]
	Don’t know	11.9	9.6	9.9	7.9	−1.7	[−3.4 ; -0.1]
Internet	Agree	7.9	1.8	9.9	2.3	0.5	[−2.7 ; 3.7]
	Undecided	12.0	12.4	17.6	18.7	6.3	[−45.7 ; 58.2]
	Disagree	64.0	71.0	58.2	65.8	−5.2	[−52.4 ; 42.1]
	Don’t know	16.1	14.8	14.3	13.2	−1.6	[−4.6 ; 1.4]
Do something against it oneself	Agree	29.0	28.6	49.5	49.6	21.0	[13.6 ; 28.4]
	Undecided	24.5	23.0	22.6	21.2	−1.8	[−6.2 ; 2.6]
	Disagree	38.0	40.1	21.1	22.5	−17.6	[−23.7 ; -11.4]
	Don’t know	8.5	8.4	6.8	6.6	−1.8	[−3.0 ; -0.5]

**Table 5:** Treatment recommendations for schizophrenia and major depression

		**Schizophrenia**		**Major depression**			
		**Raw %**	**Adj. %**	**Raw %**	**Adj. %**	**Change**	**95% CI**
Psychotherapy	Agree	79.2	82.7	69.9	74.6	−8.1	[−21.6; 5.3]
	Undecided	11.3	11.8	18.7	20.1	8.3	[5.9; 10.6]
	Disagree	5.2	1.4	8.0	2.1	0.7	[−0.7 ; 2.2]
	Don’t know	4.3	4.1	3.4	3.3	−0.8	[−7.2 ; 5.5]
Psychotropic medication	Agree	37.1	37.0	20.5	20.5	−16.5	[−22.5 ; -10.6]
	Undecided	20.6	20.6	21.5	21.5	0.9	[−4.2 ; 6.0]
	Disagree	32.7	32.5	47.9	47.5	15.0	[8.1 ; 21.9]
	Don’t know	9.6	9.8	10.1	10.5	0.7	[−0.2 ; 1.6]
Relaxation techniques	Agree	29.4	29.3	38.7	39.1	9.8	[3.5 ; 16.1]
	Undecided	27.7	27.1	29.8	29.1	2.0	[−3.6 ; 7.7]
	Disagree	29.0	31.6	19.2	21.1	−10.5	[−16.4 ; -4.5]
	Don’t know	13.9	12.0	12.3	10.6	−1.4	[−1.9 ; -0.8]
Meditation/Yoga	Agree	31.7	32.1	42.2	42.9	10.8	[3.9 ; 17.6]
	Undecided	29.9	28.7	30.6	29.4	0.7	[−4.6 ; 5.9]
	Disagree	28.3	30.3	20.8	22.3	−8.0	[−13.3 ; -2.8]
	Don’t know	10.1	8.8	6.4	5.5	−3.3	[−4.8 ; -1.9]
Natural remedies	Agree	22.4	21.4	40.9	40.6	19.2	[12.6 ; 25.8]
	Undecided	23.5	21.4	29.7	27.8	6.4	[1.1 ; 11.6]
	Disagree	43.1	45.5	22.8	24.4	−21.1	[−27.5 ; -14.6]
	Don’t know	11.0	11.7	6.6	7.2	−4.5	[−5.8 ; -3.2]
Acupuncture	Agree	20.7	19.5	32.0	30.9	11.4	[3.8 ; 18.9]
	Undecided	25.8	24.4	27.6	26.5	2.1	[−4.2 ; 8.4]
	Disagree	38.7	41.4	27.3	29.5	−11.9	[−18.5 ; -5.4]
	Don’t know	14.8	14.7	13.1	13.2	−1.5	[−5.2 ; 2.2]

**Table 6:** Stereotypes of schizophrenia and major depression

		**Schizophrenia**		**Major depression**			
		**Raw %**	**Adj. %**	**Raw %**	**Adj. %**	**Change**	**95% CI**
The person is unpredictable	Agree	72.6	73.6	34.5	35.3	−38.3	[−42.9 ; -33.8]
	Undecided	19.3	18.1	35.1	33.4	15.3	[10.9 ; 19.6]
	Disagree	8.1	8.3	30.4	31.3	23.0	[−11.1 ; 57.2]
The person is dangerous	Agree	36.1	38.3	14.5	15.2	−23.1	[−27.6 ; -18.6]
	Undecided	32.4	29.2	23.2	20.8	−8.4	[−12.7 ; -4.2]
	Disagree	31.5	32.5	62.3	64.0	31.5	[27.6 ; 35.5]
The person is responsible for his/her condition	Agree	6.7	1.2	8.7	1.6	0.4	[−20.0 ; 20.8]
	Undecided	23.5	25.0	29.4	31.9	6.9	[−5.1 ; 18.9]
	Disagree	69.8	73.8	61.9	66.5	−7.3	[−55.6 ; 41.0]
Even with treatment, the person’s state will not change significantly	Agree	7.0	6.1	7.4	6.4	0.3	[−1.9 ; 2.5]
	Undecided	23.2	23.7	22.6	23.0	−0.7	[−4.9 ; 3.5]
	Disagree	69.8	70.2	70.0	70.6	0.4	[−3.4 ; 4.2]

**Table 7:** Separation from persons with schizophrenia and major depression

		**Schizophrenia**		**Major depression**			
		**Raw %**	**Adj. %**	**Raw %**	**Adj. %**	**Change**	**95% CI**
This person is different from others	Agree	38.2	32.8	13.0	10.3	−22.5	[−27.0 ; -18.1]
	Undecided	33.1	35.3	23.1	22.6	−12.7	[−17.5 ; -7.8]
	Disagree	28.6	31.9	63.9	67.1	35.2	[31.3 ; 39.1]
Basically we are all sometimes like this person. It’s just a question how pronounced this state is	Agree	28.5	29.7	58.2	59.1	29.4	[24.7 ; 34.2]
	Undecided	29.5	30.6	26.9	27.1	−3.5	[−8.1 ; 1.2]
	Disagree	42.0	39.8	14.9	13.7	−26.1	[−28.8 ; -23.2]

**Table 8:** Emotional reactions to persons with schizophrenia or major depression

		**Schizophrenia**		**Major depression**			
		**Raw %**	**Adj. %**	**Raw %**	**Adj. %**	**Change**	**95% CI**
I feel the need to help him/her	Agree	72.1	74.3	80.9	82.5	8.2	[4.1 ; 12.4]
	Undecided	22.4	24.5	15.6	16.7	−7.8	[−11.9 ; -3.7]
	Disagree	5.5	1.2	3.5	0.8	−0.4	NA
I feel pity for him/her	Agree	70.9	70.9	79.1	79.5	8.6	[4.3 ; 12.9]
	Undecided	19.4	19.1	16.1	15.7	−3.4	[−7.1 ; 0.3]
	Disagree	9.7	10.0	4.8	4.8	−5.2	[−11.4 ; 1.0]
I feel sympathy for him/her	Agree	46.4	46.4	69.5	69.9	23.5	[18.8 ; 28.3]
	Undecided	38.2	37.6	23.5	22.9	−14.7	[−19.2 ; -10.1]
	Disagree	15.4	16.0	7.0	7.2	−8.8	[−15.2 ; -2.5]
I feel uncomfortable	Agree	57.9	59.7	35.2	36.8	−22.9	[−27.7 ; -18.0]
	Undecided	23.5	22.6	25.8	25.2	2.6	[−1.6 ; 6.8]
	Disagree	18.6	17.7	39.0	37.9	20.2	[13.9 ; 26.5]
He/she makes me feel insecure	Agree	48.0	49.1	29.4	30.6	−18.5	[−23.3 ; -13.7]
	Undecided	25.5	26.5	28.9	30.6	4.1	[−0.5 ; 8.6]
	Disagree	26.5	24.4	41.7	38.8	14.4	[8.7 ; 20.2]
He/she scares me	Agree	35.4	35.3	22.6	22.3	−13.0	[−17.5 ; -8.5]
	Undecided	33.2	32.9	21.9	21.5	−11.4	[−15.8 ; -7.0]
	Disagree	31.4	31.8	55.5	56.2	24.4	[20.3 ; 28.5]
I feel annoyed by him/her	Agree	6.5	6.4	7.4	7.1	0.7	[−1.7 ; 3.2]
	Undecided	17.5	18.3	14.5	15.1	−3.2	[−7.0 ; 0.5]
	Disagree	76.0	75.3	78.1	77.8	2.5	[−1.0 ; 5.9]
I react angrily	Agree	5.1	3.7	6.1	4.4	0.7	[−1.0 ; 2.3]
	Undecided	13.3	12.8	13.4	12.9	0.1	[−3.1 ; 3.4]
	Disagree	81.6	83.5	80.5	82.7	−0.8	[−4.0 ; 2.5]
I am amused by something like that	Agree	3.1	0.5	1.3	0.2	−0.3	[−17.0 ; 16.3]
	Undecided	7.8	6.2	3.1	2.4	−3.8	[−9.1 ; 1.5]
	Disagree	69.1	93.3	95.6	97.4	4.1	[−29.7 ; 38.0]

**Table 9:** Desire for social distance from persons with schizophrenia and major depression

		**Schizophrenia**		**Major depression**			
		**Raw %**	**Adj. %**	**Raw %**	**Adj. %**	**Change**	**95%CI**
Work together	Accept	41.7	38.0	47.6	43.8	5.8	[1.0 ; 10.6]
	Undecided	37.8	39.8	37.0	39.3	−0.5	[−5.4 ; 4.4]
	Reject	20.5	22.2	15.4	16.9	−5.3	[−0.7 ; 12.4]
Have as neighbor	Accept	32.7	29.1	50.4	46.5	17.4	[12.8 ; 22.1]
	Undecided	40.5	42.1	36.4	38.9	−3.2	[−8.1 ; 1.8]
	Reject	26.8	28.8	13.2	14.6	−14.2	[−21.2 ; -7.2]
Introduce to a friend	Accept	25.6	22.2	39.1	35.2	13.0	[8.6 ; 17.3]
	Undecided	35.5	36.3	37.9	39.8	3.5	[−1.3 ; 8.4]
	Reject	38.9	41.5	23.0	25.0	−16.5	[−21.0 ; -12.0]
Marry into family	Accept	12.2	10.3	21.1	18.2	7.9	[4.5 ; 11.4]
	Undecided	33.5	33.9	40.1	41.4	7.5	[2.7 ; 12.4]
	Reject	54.3	55.8	38.8	40.3	−15.5	[−19.6 ; -11.4]
Recommend for a job	Accept	11.1	11.0	18.7	18.6	7.6	[4.1 ; 11.2]
	Undecided	32.0	30.3	40.4	38.6	8.3	[3.7 ; 13.0]
	Reject	56.9	58.8	40.9	42.7	−16.1	[−20.1 ; -12.0]
Rent a room	Accept	11.6	9.9	22.1	19.6	9.7	[6.2 ; 13.1]
	Undecided	28.6	26.7	37.6	36.4	9.7	[5.2 ; 14.3]
	Reject	59.8	63.4	40.3	44.0	−19.4	[−23.5 ; -15.3]
Take care of children	Accept	1.7	1.6	9.5	9.0	7.4	[4.8 ; 10.0]
	Undecided	9.9	8.9	19.5	17.8	8.9	[5.6 ; 12.3]
	Reject	88.4	89.5	71.0	73.2	−16.3	[−20.4 ; -12.2]

## Results

### Beliefs about schizophrenia and major depressive disorder

#### Recognition

With schizophrenia, the vast majority of respondents (78%) endorsed the view that the person depicted in the vignette suffers from an illness in the medical sense, whereas with major depressive disorder this view was shared by only 58%, while 21% each answered in the negative or could not give an answer (Table 1).

#### Causal beliefs

In Table 2 the French public’s beliefs about the causes of the two mental disorders in question are reported. Biogenetic factors were twice as likely to be endorsed as a cause of schizophrenia than as a cause of major depressive disorder. With depression, the majority disagreed with this etiological explanation. In contrast, psychosocial stress was considered a cause of depression by four out five respondents, while with schizophrenia the percentage was markedly lower. Smaller or no differences between both disorders were observed with regard to childhood adversities. The same applies to personality factors, except for weak will which was made responsible considerably more frequently when the schizophrenia vignette was presented. In case of schizophrenia, ‘negative life events’ were chosen most frequently as a cause (65%), followed by ‘unconscious conflict’ (53%) and ‘disturbance of brain metabolism’ (51%). The most frequently endorsed causes of depression were the three factors representing current stress (negative life events, stress at work, and troubles in family/partnership).

#### Anticipated prognosis

In general, respondents were rather optimistic concerning the prognosis of both disorders. However, this tendency was less pronounced with schizophrenia where markedly fewer respondents explicitly disagreed with statements positing a poor course and where more respondents remained undecided or agreed with them (Table 3).

#### Help-seeking recommendations

As shown in Table 4, among the professional helpers proposed, respondents presented with the schizophrenia vignette recommended most frequently turning to a psychiatrist (81%), followed by psychotherapists (77%), and general practitioners (75%). As concerns major depressive disorder, the order was reversed, with general practitioners being more frequently recommended (83%) than psychotherapists (68%) and psychiatrists (59%). In case of major depression, turning to a practitioner of complementary medicine or making a health cure in a spa was less frequently advised against and informal self-help or asking a confidant for help was more frequently recommended. There were no differences between both disorders concerning seeking help from a priest, joining a self-help group, or visiting the Internet.

#### Treatment recommendations

Psychotherapy was the uncontested favorite for the treatment of both schizophrenia and major depressive disorder. As concerns schizophrenia, psychotropic medication came next. As concerns depression, medication was less likely to be recommended than all other treatment options; while psychotropic medication was endorsed by only 20% of the respondents, relaxation techniques, meditation/yoga, natural remedies, or acupuncture were endorsed by 32% - 42%. More respondents recommended medication for the treatment of schizophrenia than advised against it, with depression the reverse was found (Table 5).

### Attitudes towards people with schizophrenia and major depressive disorder

#### Stereotypes

Among the four stereotypes, the perception that the person depicted in the vignette is unpredictable was most frequently endorsed, followed by the perception of dangerousness. Both were twice as frequently associated with schizophrenia (73% and 34%, respectively) than with major depressive disorder (36% and 14%, respectively). The remaining two stereotypes, namely that the person is responsible for his/her condition and that the condition is hard to treat, were shared each on their own by less than ten percent of respondents. This applied equally to schizophrenia and major depressive disorder (Table 6).

#### Separation of ‘us’ from ‘them’

As shown in Table 7, respondents reacted quite differently to the two vignettes. In the case of major depressive disorder, the tendency to separate oneself from the person in the vignette was much less pronounced. As compared to schizophrenia, respondents disagreed over twice as frequently with the statement that ‘the person is different from others’ (64% vs. 29%), and agreed over twice as frequently with the statement that ‘basically we are all sometimes like this person’ and that ‘it is only a question how pronounced this state is’.

#### Emotional reactions

In Table 8, the emotional reactions of respondents to persons with schizophrenia or major depressive disorder are presented. In general, respondents reacted most frequently with pro-social feelings (need to help, pity, sympathy), followed by fear and related feelings (uncomfortable, insecure), whereas feelings of anger, annoyance, and amusement were elicited only rarely. However, the schizophrenia vignette evoked markedly more fear than the depression vignette. While, for instance, 58% of respondents felt uncomfortable with the person displaying symptoms of schizophrenia, with major depressive disorder the same feeling was expressed by only 35%. In contrast, more pro-social feelings were expressed when respondents were presented with the depression vignette, although the difference between both disorders here was less pronounced. As concerns feelings of anger, annoyance, or amusement, no differences existed between both disorders.

#### Desire for social distance

As shown in Table 9, across all seven social relationships, respondents distanced themselves more strongly from the person with symptoms of schizophrenia than from the person with symptoms of major depressive disorder. Except for the most distant relationships (colleague at work, neighbor, introducing to friends), where a considerable proportion did accept the person with schizophrenia or was at least undecided in this regard, over half of respondents rejected him or her in closer relationships. With the depressive person the amount of rejection equaled that of indecisiveness. The vast majority of respondents were opposed to letting the person take care of children no matter which vignette had been presented.

## Discussion

### Beliefs about schizophrenia and major depressive disorder

A clear pattern emerges from our findings: Schizophrenic symptoms are defined by the vast majority of respondents as expression of mental illness, caused by biogenetic factors and current stress. In contrast, depressive symptoms are less unanimously defined as mental illness, and current stress plays here a dominant causal role. The prognosis of major depressive disorder appears more favorable than that of schizophrenia. In the eyes of the public, psychiatrists represent the most appropriate helping source for people suffering from schizophrenic symptoms, and, apart from psychotherapy, medication is recommended most frequently for treatment. For depressive symptoms, general practitioners are considered most frequently as a source for help; taking a health cure or seeing a practitioner in complementary medicine are less likely to be rejected; apart from formal help, there is also a strong emphasis on self-help and social support. In the eyes of the public, relaxation techniques and ‘alternative’ methods are better suited for the treatment of major depression than psychotropic medication. Thus, although there exists a certain overlap between both disorders, the French public seems to draw a clear line between schizophrenia and major depressive disorder: on one hand schizophrenia as an illness with a strong biological component, demanding psychiatric treatment, on the other hand major depression which is seen mainly as a consequence of the exposure to psycho-social stress, benefitting also from alternative treatments.

In 2011, a similar survey had been conducted in Germany, using the same interview [12]. Since the interview mode was different (in France on-line, in Germany face-to-face) a direct comparison of results for schizophrenia and depression between both studies seems problematic [19]. However, it is legitimate to examine within each study the differences between both disorders and then contrast them across studies [20]. Apart from many similarities, there were some interesting differences. The divide between both disorders regarding the endorsement of current stress as a cause was more pronounced in France than in Germany. For instance, in France work-related stress was seen as a cause of depression by 80% and as a cause of schizophrenia by only 47%, while in Germany the respective percentages were 79% and 62%; thus, the difference between both disorders was larger in France than in Germany. Moreover, the difference between both disorders in relying on self-help was more pronounced in France (depression 50%, schizophrenia 29%) than in Germany (depression 59%, schizophrenia 47%). Finally, while in France ‘alternative’ methods like natural remedies or acupuncture were more frequently recommended for the treatment of depression (41% and 32%, respectively) than for the treatment of schizophrenia (22% and 21%, respectively), in Germany practically no difference was found between both disorders (27%/24% and 18%/17%) [12]. All these results suggest that the distinction made between schizophrenia and depression tends to be more marked in France than in Germany.

Irrespectively of the type of disorder, psychotherapy was the clear favorite. The acceptance of it was even more pronounced regarding the treatment of schizophrenia, which appears somewhat counterintuitive. This is not a French specialty. In the afore-mentioned German survey, 73% of respondents recommended psychotherapy for the treatment of depression and 84% for the treatment of schizophrenia. Similar findings have also been reported from previous studies (e.g. [21-23]). The public’s reservation against psychotropic medication has also been observed in Germany. However, the contrast to the acceptance of psychotherapy was not as marked there as in France. For instance concerning the treatment of schizophrenia, 79% of respondents opted in France for psychotherapy and only 37% for medication, whereas in Germany the corresponding figures were 84% and 51%. A similar pattern was also found for depression (70%/21% versus 73%/36%). Interestingly, the difference between both countries in attitudes does not correspond with the actual use of psychotropic medication which is higher in France than in Germany [24]. The reasons for the aversion against psychotropic medication expressed by the French public are certainly manifold. One may be that while in both countries current stress was in sum more frequently endorsed than biogenetic causes, the preponderance of the first was slightly more pronounced in France. Regarding depression, for instance, the ratio between the sum of stress factors and the sum of biogenetic causes was twice as high in France as in Germany. Another reason may be that medication still tends to be seen by the public less as treatment of the real causes of an illness than psychotherapy, particularly psychoanalysis, which in France still enjoys relatively great popularity [25]. Finally, the widespread fear of getting addicted to medication may play a role, as the public is not able to sufficiently distinguish between drugs that have this unwanted effect, such as benzodiazepines, and others, such as antidepressants or antipsychotics, which do not have it [26].

### Attitudes towards people with schizophrenia and major depressive disorder

Across all components studied, attitudes towards people with schizophrenia were more unfavorable than those towards people with major depressive disorder. People with schizophrenia more frequently were perceived as unpredictable and dangerous, there was a stronger need to separate ‘us’ from ‘them’ , they were more frequently met with fear and less frequently reacted to with pro-social feelings, and they faced more rejection. As with beliefs, the comparison with the German survey yields some interesting differences. For instance, concerning the endorsement of dangerousness, the discrepancy between both disorders was greater in France than in Germany (ratio schizophrenia/major depression in France 36%/14%, in Germany 23%/19%). While in France, 28% of respondents agreed with the statement that ‘we are all sometimes like this person’ in case of schizophrenia and 58% in case of major depression, in Germany, the difference between both disorders was smaller with 26% and 44%, respectively. While the difference between schizophrenia and major depression in the amount of fear expressed by respondents was in both countries the same, the difference in pro-social feelings in favor of major depression was more marked in France. Taken together, the discrepancy between attitudes towards people suffering from schizophrenia and major depressive disorder appears to be slightly more pronounced in France than in Germany. As already observed with illness beliefs [6], French people seem to distinguish between both disorders even more clearly than their German counterparts. While depression is perceived as something most people are familiar with through personal experience and as something which more or less belongs to normal life, people with schizophrenia are perceived as strange (by 60% of respondents as compared to only 16% with depression) and their behavior appears as incomprehensible (45% versus 21%). Another indication of the great familiarity of the French people with the notion of depression may be that almost all respondents (88%) identified the depressive symptoms depicted in the vignette as an expression of some sort of depression [27].

### Limitations

Before concluding, some limitations of our study should be mentioned. First, the focus on attitudes may be looked upon as a limitation since it allows predicting behavior with less than ideal accuracy. However, rather than using them as a proxy for individual behaviors, public attitudes can also be conceptualized at a collective level as a reflection of cultural conceptions of mental illness. Such conceptions form a cultural context that influences the way we behave towards those suffering from mental illness. As Link et al. [28] have pointed out, ‘as a context this cultural conception becomes an external reality, something that individuals must take into account when they make decisions and enact behavior’ (p. 255). Second, due to selection processes typical for on-line surveys [19], the representativeness of the results for the whole of the French population may be considered uncertain. In this context it seems worth noting that the feasibility of this interview mode for exploring beliefs and attitudes about mental disorders has recently been shown in another survey in France [29]. Third, social desirability may have biased our findings in the sense that respondents may have responded to interview questions in a way that makes them appear socially desirable, and may not actually have responded according to their true reactions. However, this seems to be less a problem in online interviews than in face-to-face interviews [30]. Finally, we do not know how the terms used in the interview were understood by the respondents. For instance, what the lay public associates with this term ‘psychotherapy’ does not necessarily reflect how psychotherapy is defined by mental health professionals. A recently published study comes to the conclusion that lay people appear to have ‘a modestly realistic but somewhat naïve view of the process and efficacy of psychotherapy’ [31]. It is not unlikely that many people simply associate with psychotherapy talking with patients [32].

## Conclusions

In conclusion, we can state that there is a strong tendency among the French public to make a distinction between schizophrenia and major depression concerning illness beliefs. Thus, despite theoretical and methodological differences, our results converge with those reported by Roelandt et al. [1,2]. What they found exploring the social representations of mundane labels for mental illness (‘insane’ , ‘depressive’) resonates in our findings based on descriptions of schizophrenia and major depressive disorder as they are defined by modern psychiatric diagnostic classifications. This applies equally to beliefs about these illnesses (e.g., their causes or treatment) as to attitudes towards persons suffering from them. There is a need for interventions trying to reduce existing misconceptions in order to improve the care of patients.

## Competing interests

All authors have no competing interests to declare.

## Authors’ contributions

MCA developed the interview and drafted the manuscript. AM and CR were involved in the translation of the interview and helped coordinate the survey. AM and TR performed the statistical analyses. MT conceived of the study, organized the survey, and helped draft the manuscript. All authors read and approved the final manuscript.

## Pre-publication history

The pre-publication history for this paper can be accessed here:

http://www.biomedcentral.com/1471-244X/13/313/prepub
